# Altered Topological Organization of White Matter Structural Networks in Patients with Neuromyelitis Optica

**DOI:** 10.1371/journal.pone.0048846

**Published:** 2012-11-07

**Authors:** Yaou Liu, Yunyun Duan, Yong He, Jun Wang, Mingrui Xia, Chunshui Yu, Huiqing Dong, Jing Ye, Helmut Butzkueven, Kuncheng Li, Ni Shu

**Affiliations:** 1 Department of Radiology, Xuanwu Hospital, Capital Medical University, Beijing, P.R. China; 2 State Key Laboratory of Cognitive Neuroscience and Learning, Beijing Normal University, Beijing, P.R. China; 3 Department of Neurology, Xuanwu Hospital, Capital Medical University, Beijing, P.R. China; 4 Department of Medicine, University of Melbourne, Parkville, Australia; Kyushu University, Japan

## Abstract

**Objective:**

To investigate the topological alterations of the whole-brain white-matter (WM) structural networks in patients with neuromyelitis optica (NMO).

**Methods:**

The present study involved 26 NMO patients and 26 age- and sex-matched healthy controls. WM structural connectivity in each participant was imaged with diffusion-weighted MRI and represented in terms of a connectivity matrix using deterministic tractography method. Graph theory-based analyses were then performed for the characterization of brain network properties. A multiple linear regression analysis was performed on each network metric between the NMO and control groups.

**Results:**

The NMO patients exhibited abnormal small-world network properties, as indicated by increased normalized characteristic path length, increased normalized clustering and increased small-worldness. Furthermore, largely similar hub distributions of the WM structural networks were observed between NMO patients and healthy controls. However, regional efficiency in several brain areas of NMO patients was significantly reduced, which were mainly distributed in the default-mode, sensorimotor and visual systems. Furthermore, we have observed increased regional efficiency in a few brain regions such as the orbital parts of the superior and middle frontal and fusiform gyri.

**Conclusion:**

Although the NMO patients in this study had no discernible white matter T2 lesions in the brain, we hypothesize that the disrupted topological organization of WM networks provides additional evidence for subtle, widespread cerebral WM pathology in NMO.

## Introduction

Neuromyelitis optica (Devic’s disease) (NMO) is an inflammatory, demyelinating syndrome of the central nervous system characterized by severe attacks of optic neuritis and myelitis [1], [2]. Imaging evidence of brain involvement in NMO has been demonstrated by several recent studies [3]–[8], challenging the notion of spinal cord and optic nerve restricted pathology in NMO.

Diffusion tensor imaging (DTI) is a powerful, non-invasive imaging technique which has the potential to map the white matter (WM) integrity and anatomical connectivity of the human brain *in vivo* [9]–[11]. Several previous studies have utilized the DTI technique and diffusion measures to investigate changes in the brain WM in patients with NMO. For example, Rocca and colleagues examined the alteration of mean diffusivity histogram-derived metrics of the normal appearing white matter (NAWM), and found that there were no significant differences between the NMO patients and controls [3]. In contrast, our group showed that NMO patients had abnormal diffusion indices in the cerebral corticospinal tract and optic radiation, potentially suggesting transsynaptic neural degeneration arising from spinal and optic nerve lesions [6]. Additionally, our group has recently shown more widespread, subtle cerebral abnormalities in NMO patients, utilizing the tract based spatial statistics technique [8]. However, it is unknown whether these abnormalities are significant enough to alter the brain anatomical connectional architecture, as assessed by whole-brain structural network analysis.

Recent studies have suggested that the structural networks of the human brain can be constructed by using diffusion MRI and tractography methods [for reviews, see 12,13]. In healthy populations, the WM structural networks have been mapped by using deterministic [14]–[17] or probabilistic [18]–[20] tractography methods. Importantly, these studies have consistently revealed that the human WM structural networks have many non-trivial topological properties such as the small-worldness [high local clustering and short path length linking individual nodes [21]], high efficiency at a low wiring cost and network hubs in the posterior medial cortical regions (e.g., the precuneus). These results are largely compatible with previous morphological and functional brain network studies [22]–[26]. Recently, these diffusion MRI-based methods have been applied to investigate the topological changes in normal aging [20], Alzheimer’s disease [27], schizophrenia [28], early onset blindness [29], and multiple sclerosis [30] demonstrating specific alterations in the human brain connectome in diseased populations [13], [31].

In this study, we employed diffusion tensor tractography and graph theoretical approaches to assess connectional architecture of whole-brain WM structural networks in NMO patients. To our knowledge, the present study is the first to explore the topological alterations of diffusion MRI-derived network in patients with NMO, seeking to clarify the extent and potential structural consequences of cerebral pathology in an illness not associated with overt WM changes visible on conventional MRI sequences such as T2-weighted, T1-weighted or FLAIR sequences.

## Materials and Methods

### Participants

This study included 26 NMO patients (24 females; mean age 35.7±11.9 years) and 26 age and sex-matched healthy controls (24 females; mean age 34.1±10.1 years). The diagnosis of NMO was made by an experienced neurologist with special expertise in white matter disease (Dr. Jing Ye). All patients fulfilled the recently revised diagnostic criteria for NMO [1]. As NMO IgG testing was not available at our hospital when the patients were assessed and scanned, all patients met both the absolute criteria, namely episodes of optic neuritis and myelitis, as well as the supporting criteria of brain MRI results that were negative or nondiagnostic for multiple sclerosis, and MRI evidence of a longitudinally extensive spinal cord T2 lesion spanning at least three vertebral segments. To improve diagnostic certainty, we asked the patients in this study to be tested for serum anti-AQP4 antibodies when the test became available to us in 2010. To date, 18 of the studied patients were tested and 16 patients (89%) were anti-AQP4 positive, further confirming that our included patients are similar to other reported NMO cohorts. The main demographic and clinical characteristics of the patients are reported in Table 1. Twenty-one patients had no T2 WM lesions on brain MRIs and five of them had small, non-specific WM lesions. None of the participating patients had been treated with medications for NMO (e.g., corticosteroids and immunosuppressants) within 3 months before MRI scanning. This study was approved by the institutional review board of Xuanwu Hospital, and written informed consent was obtained from each participant.

**Table 1:** Demographics and clinical characteristics of all participants.

Characteristics	NMO Patients(n = 26)	Controls(n = 26)	*P-*value
Mean age ± std (range)[years]	35.7±11.9(19–59)	34.1±10.1(19–52)	0.67
Gender (M/F)	2/24	2/24	>0.99
Median EDSS (range)	3.0 (1.0–6.0)	–	–
Median disease duration(range) [months]	48 (6–240)	–	–

Abbreviations: NMO: neuromyelitis optica; EDSS: expanded disability status scale. See text for further details.

### Image Acquisition

All participants were scanned with a 1.5T MRI scanner (Sonata, Siemens Medical Systems, Erlangen, Germany). T2, T1 and DTI images were acquired with the following sequences: (a) T2-weighted turbo spin echo imaging [repetition time (TR)/echo time (TE) = 5460/94 ms; number of excitation (NEX) = 3; echo train length = 11; matrix = 224×256; field of view (FOV) = 210 mm×240 mm; slice = 30; slice thickness = 4 mm; orientation  =  axial], (b) T1-weighted spin echo imaging [TR/TE = 1900/4 ms; NEX = 1; matrix = 224×256; FOV = 220 mm×250 mm; slices = 96; slice thickness = 1.7 mm; orientation  =  sagittal] and (c) spin-echo single-shot echo planar imaging (EPI) [TR/TE = 5000/100 ms; NEX = 10; matrix = 128×128; FOV = 240 mm×240 mm; slices = 30; slice thickness = 4 mm; slice gap = 0.4 mm; orientation  =  axial; 6 nonlinear diffusion weighting gradient directions with b = 1000 s/mm^2^ and 1 additional image without diffusion weighting (i.e., b = 0 s/mm^2^)].

### Data Preprocessing

Eddy current distortions and motion artifacts in the DTI dataset were corrected by applying affine alignment of each diffusion-weighted image to the b = 0 image, using FMRIB’s Diffusion Toolbox (FSL, version 3.3; www.fmrib.ox.ac.uk/fsl). After this process, the diffusion tensor elements were estimated by solving the Stejskal and Tanner equation [11], [32], and then the reconstructed tensor matrix was diagonalized to obtain three eigenvalues (λ_1_, λ_2_, λ_3_) and eigenvectors. The fractional anisotropy (FA) of each voxel was also calculated.

### White Matter Tractography

Diffusion tensor tractography (DTT) was implemented with DTIstudio, Version 2.40 software (H. Jiang, S. Mori; Johns Hopkins University), by using the “fiber assignment by continuous tracking” method [33]. All tracts in the dataset were computed by seeding each voxel with FA greater than 0.2. Tractography was terminated if it turned an angle greater than 45 degrees or reached a voxel with FA less than 0.2 [34].

### Network Construction

Nodes and edges are the two basic elements of a network. In this study, we defined all of the network nodes and edges using the following procedures.

#### Network node definition

**Figure 1:**
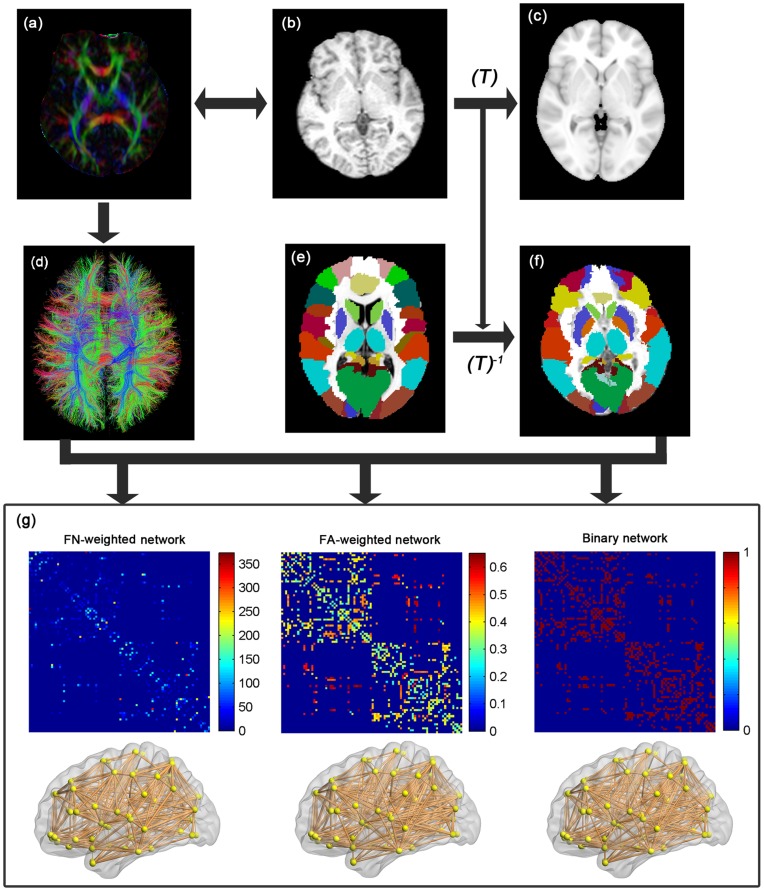
A flowchart for the construction of WM structural network by DTI. (1) The rigid coregistration from the T1-weighted structural MRI (b) to DTI native space (a) (DTI color-coded map; red: left to right; green: anterior to posterior; blue: inferior to superior) for each subject. (2) The nonlinear registration from the resultant structural MRI to the ICBM152 T1 template in the MNI space (c), resulting in a nonlinear transformation (T). (3) The application of the inverse transformation (T^−1^) to the AAL template in the MNI space (e), resulting in the subject-specific AAL mask in the DTI native space (f). All registrations were implemented in the SPM8 package. (4) The reconstruction of all of the WM fibers (d) in the whole brain using DTI deterministic tractography in DTIstudio. (5) The weighted networks of each subject (g) were created by computing the fiber numbers (FN-weighted) and the mean FA values (FA-weighted) of the fiber bundles that connected each pair of brain regions. The binary network was created by considering the existence/absence of fiber bundles between two regions. The matrices and 3D representations (lateral view) of the three kinds of WM structural networks of a representative healthy subject were shown in the bottom panel. The nodes are located according to their centroid stereotaxic coordinates, and the edges are coded according to their connection weights. For details, see the Materials and Methods section.

The procedure of defining the nodes has been previously described [20], [29] and was performed in the present study using the SPM8. Briefly, individual T1-weighted images were coregistered to the b0 images in the DTI space. The transformed T1 images were then nonlinearly transformed to the ICBM152 T1 template in the MNI space. The inverse transformations were used to warp the automated anatomical labeling (AAL) atlas [35] from the MNI space to the DTI native space. Of note, discrete labeling values were preserved by the use of a nearest-neighbor interpolation method. Using this procedure, we obtained 90 cortical and subcortical regions (45 for each hemisphere, see Table 2), each representing a node of the network (Figure 1).

**Table 2:** Cortical and subcortical regions of interest defined in the study.

Index	Regions	Abbr.	Index	Regions	Abbr.
(1,2)	Precental gyrus	PreCG	(47,48)	Lingual gyrus	LING
(3,4)	Superior frontal gyrus, dorsolateral	SFGdor	(49,50)	Superior occipital gyrus	SOG
(5,6)	Superior frontal gyrus, orbital part	ORBsup	(51,52)	Middle occipital gyrus	MOG
(7,8)	Middle frontal gyrus	MFG	(53,54)	Inferior occipital gyrus	IOG
(9, 10)	Middle frontal gyrus, orbital part	ORBmid	(55,56)	Fusiform gyrus	FFG
(11,12)	Inferior frontal gyrus, opercular part	IFGoperc	(57,58)	Postcentral gyrus	PoCG
(13,14)	Inferior frontal gyrus, triangular part	IFGtriang	(59,60)	Superior parietal gyrus	SPG
(15,16)	Inferior frontal gyrus, orbital part	ORBinf	(61,62)	Inferior parietal, but supramarginal and angular gyri	IPL
(17,18)	Rolandic operculum	ROL	(63,64)	Supramarginal gyrus	SMG
(19,20)	Supplementary motor area	SMA	(65,66)	Angular gyrus	ANG
(21,22)	Olfactory cortex	OLF	(67,68)	Precuneus	PCUN
(23,24)	Superior frontal gyrus, medial	SFGmed	(69,70)	Paracentral lobule	PCL
(25,26)	Superior frontal gyrus, medial orbital	ORBsupmed	(71,72)	Caudate nucleus	CAU
(27,28)	Gyrus rectus	REC	(73,74)	Lenticular nucleus, putamen	PUT
(29,30)	Insula	INS	(75,76)	Lenticular nucleus, pallidum	PAL
(31,32)	Anterior cingulate and paracingulate gyri	ACG	(77,78)	Thalamus	THA
(33,34)	Median cingulate and paracingulate gyri	DCG	(79,80)	Heschl gyrus	HES
(35,36)	Posterior cingulate gyrus	PCG	(81,82)	Superior temporal gyrus	STG
(37,38)	Hippocampus	HIP	(83,84)	Temporal pole: superior temporal gyrus	TPOsup
(39,40)	Parahippocampal gyrus	PHG	(85,86)	Middle temporal gyrus	MTG
(41,42)	Amygdala	AMYG	(87,88)	Temporal pole: middle temporal gyrus	TPOmid
(43,44)	Calcarine fissure and surrounding cortex	CAL	(89,90)	Inferior temporal gyrus	ITG
(45,46)	Cuneus	CUN			

Note: The regions are listed in terms of a prior template of an AAL-atlas (Tzourio-Mazoyer et al., 2002).

#### Network edge definition

To define the network edges, we selected a threshold value for the fiber bundles. Two regions were considered structurally connected at least three fibers with two end-points were located in these two regions [29]. Such a threshold selection reduced the risk of false-positive connections due to noise or the limitations in the deterministic tractography, and simultaneously ensured the size of the largest connected component (i.e., 90) in the networks was observed across all of the controls [29]. In the present study, we also evaluated the effects of different thresholds on the network analysis by setting threshold values of the number of fiber bundles that ranged from 1 to 5. We found that this thresholding procedure did not significantly influence our results. After defining the network edges, both the weighted and unweighted network analyses were performed. For the weighted networks, we defined the fiber-number (FN) and the mean FA values of the connected fibers between two regions as the weights of the network edges. For the unweighted networks, we considered the existence/absence of fiber bundles in which the network edges were defined as 1 if the fiber number between the two regions was larger than the threshold (T = 3 in our case) and as 0 otherwise. As a result, for each participant, there were three different kinds of WM networks (FN-weighted, FA-weighted and binary), each of which was represented by a symmetric 90×90 matrix.

### Network Analysis

To characterize the WM structural network, several key measures describing specific attributes of network organization were considered: network strength, global efficiency, local efficiency, shortest path length, clustering coefficient and small-worldness [36]. Here, we provide formal definitions of these network properties as utilized in our analysis.

#### Network strength

For a network (graph) G with N nodes and K edges, we calculated the strength of G as:
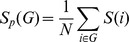
where S(i) is the sum of the edge weights *w_ij_* (*w_ij_* are the FN or FA values for the weighted networks and 1 for the binary networks) linking to node i. The strength of a network is the average of the strengths across all of the nodes in the network.

#### Small-world properties

Small-world network parameters (clustering coefficient, *C*_p_, and shortest path length, *L*_p_) were originally proposed by Watts and Strogatz [21].

In this study, we investigated the small-world properties of the weighted brain networks. The clustering coefficient of a node *i*, *C*(*i*), which was defined as the likelihood whether the neighborhoods were connected with each other or not [37], is expressed as follows:
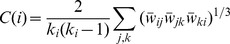
where *k_i_* is the degree of node *i*, and 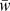 is the weight, which is scaled by the mean of all weights to control each participant’s cost at the same level. The clustering coefficient is zero, *C*(*i*) = 0, if the nodes are isolated or with just one connection, i.e., *k_i_* = 0 or *k_i_* = 1. The clustering coefficient, *C*_p_, of a network is the average of the clustering coefficient over all nodes, which indicates the extent of local interconnectivity or cliquishness in a network [21].

The path length between any pair of nodes (e.g., node *i* and node *j*) is defined as the sum of the edge lengths along this path. For weighted networks, the length of each edge was assigned by computing the reciprocal of the edge weight, 1/*w_ij_*. The shortest path length, *L_ij_*, is defined as the length of the path for node *i* and node *j* with the shortest length. The shortest path length of a network is computed as follows:
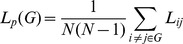
where *N* is the number of nodes in the network. The *L*_p_ of a network quantifies the ability for information propagation in parallel.

To examine the small-world properties, the clustering coefficient, *C*_p_, and shortest path length, *L*_p_, of the brain networks were compared with those of random networks. In this study, we generated 100 matched random networks, which had the same number of nodes, edges, and degree distribution as the real networks [38]. Of note, we retained the weight of each edge during the randomization procedure such that the weight distribution of the network was preserved. Furthermore, we computed the normalized shortest path length, 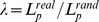, and the normalized clustering coefficient, 
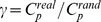
, where 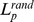 and 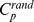 are the mean clustering coefficient and the mean shortest path length of 100 matched random networks. Of note, the two parameters correct the differences in the edge number and degree distribution of the networks across individuals. A real network would be considered small-world if 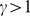 and 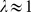 [21]. In other words, a small-world network has not only the higher local interconnectivity but also the approximately equivalent shortest path length compared with the random networks. These two measurements can be summarized into a simple quantitative metric, small-worldness, 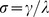, which is typically greater than 1 for small-world networks [39].

#### Network efficiency

The global efficiency of G measures the global efficiency of the parallel information transfer in the network [40], which can be computed as [40]:
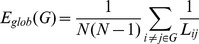
where L_ij_ is the shortest path length between node i and node j in G.

The local efficiency of G reveals how much the network is fault tolerant, showing how efficient the communication is among the first neighbors of the node i when it is removed. The local efficiency of a graph is defined as [40]:
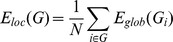
where G_i_ denotes the subgraph composed of the nearest neighbors of node i.

#### Regional nodal characteristics

To determine the nodal (regional) characteristics of the WM networks, we computed the regional efficiency, E_nodal_(i) [41]:
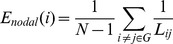
where L_ij_ is the shortest path length between node i and node j in G. E_nodal_(i) measures the average shortest path length between a given node i and all of the other nodes in the network. The node i was considered a brain hub if E_nodal_(i) was at least one standard deviation (SD) greater than the average nodal efficiency of the network (i.e., E_nodal_(i) > mean±SD).

### Statistical Analysis

To determine whether the network topology in NMO was significantly changed, a general linear model (GLM) was then conducted to test group differences on the global and regional network parameters while removing the effects of age and gender.

## Results

In the present study, we constructed three different kinds of networks for each participant, including FN-weighted, FA-weighted and binary networks (Figure 1). Despite the different connectivity metrics of the networks, we observed compatible results for the group differences. In the present study, we focused mainly on the results that were obtained from the analyses of the FN-weighted networks (for the results of the FA-weighted and binary network analyses, see Figures S1 and S2, Table 3).

**Table 3:** Comparisons of global network measures between controls and NMO patients.

FN-weighted network	S_p_	E_glob_	E_loc_	L_p_	C_p_	λ	γ	σ
Control	476.07±84.66	0.59±0.04	0.91±0.07	1.71±0.12	0.35±0.02	1.14±0.04	3.86±0.31	3.39±0.28
NMO	462.88±74.16	0.57±0.05	0.89±0.07	1.77±0.17	0.35±0.02	1.16±0.04	4.16±0.49	3.57±0.37
*T*-value	−0.91	−1.59	−0.98	1.67	0.99	2.29	2.85	2.17
*P*-value	NS	NS	NS	NS	NS	0.027*	0.007*	0.035*
**FA-weighted network**	S_p_	E_glob_	E_loc_	L_p_	C_p_	λ	γ	σ
Control	4.07±0.37	0.49±0.02	0.69±0.02	2.05±0.06	0.45±0.02	1.09±0.01	3.09±0.28	2.84±0.23
NMO	3.91±0.51	0.48±0.02	0.69±0.03	2.08±0.11	0.45±0.02	1.09±0.02	3.35±0.41	3.06±0.33
*T*-value	−1.55	−1.42	−0.23	1.50	0.24	1.17	2.88	3.04
*P*-value	NS	NS	NS	NS	NS	NS	0.006*	0.004*
**Binary network**	S_p_	E_glob_	E_loc_	L_p_	C_p_	λ	γ	σ
Control	10.18±0.77	0.46±0.01	0.69±0.02	2.16±0.06	0.46±0.02	1.10±0.01	3.17±0.27	2.88±0.23
NMO	9.93±0.99	0.46±0.02	0.68±0.03	2.20±0.10	0.46±0.02	1.11±0.02	3.43±0.41	3.09±0.33
*T*-value	−1.25	−1.67	−0.26	1.72	0.14	2.05	2.92	2.94
*P*-value	NS	NS	NS	NS	NS	0.046*	0.005*	0.005*

Note: The WM network with different connectivity metrics (FN-weighted, FA-weighted and binary networks) for each participant was constructed under the threshold T = 3.

* *p*<0.05 was considered significant. NS: not significant.

### Small-world Topology of the White Matter Structural Networks

**Figure 2:**
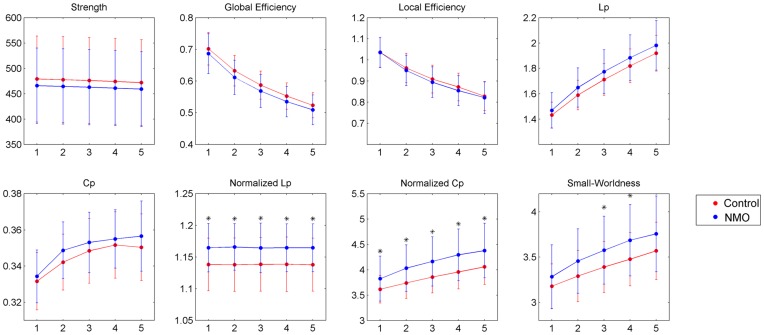
Group differences in global measures of WM structural networks were quantified between control and NMO groups. The FN-weighted network of each subject was constructed with different thresholds (T = 1,2,3,4,5). The threshold (horizontal axis) determined the minimum number of streamlines that needed to interconnect a pair of nodes for a connection to be assumed. Data points marked with a star indicate a significant difference (*p*<0.05) between groups. Significant group effects in normalized path length and normalized clustering were observed for all thresholds considered. A trend of increased small-worldness was also observed in NMO patients versus controls.

Based on the constructed WM structural network for each subjects, both controls and NMO patients showed small-world organization of the WM networks expressed by a 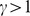 and 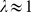 (Figure 2 and Table 3). Between the NMO and control groups, no significant differences were found in the strength, global efficiency, local efficiency, absolute path length and absolute clustering of the WM networks at *p*<0.05. However, NMO patients exhibited abnormal small-world parameters, as indicated by increased normalized path length, increased normalized clustering and increased small-worldness (Figure 2 and Table 3).

### Distribution of Altered Regional Efficiency in NMO

#### Hub regions of the white matter structural networks

**Figure 3:**
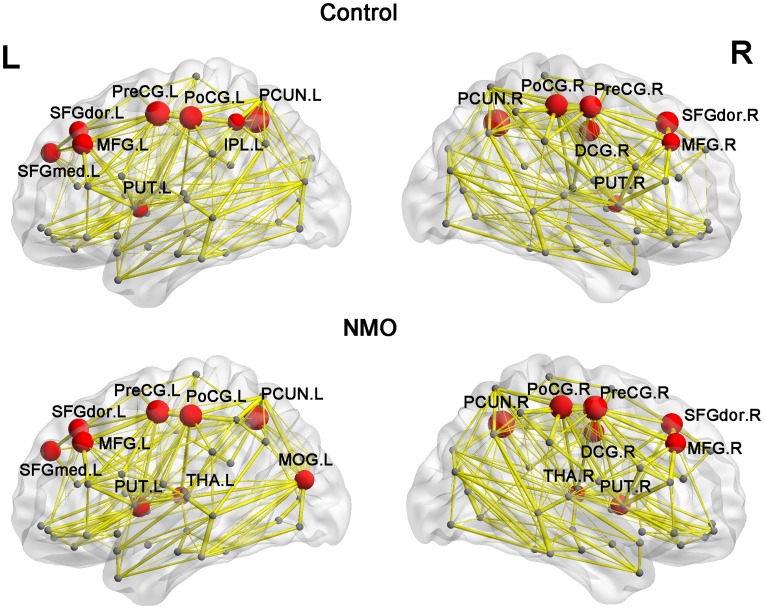
The hub region distributions of the WM structural networks in the control and NMO groups. The hub nodes are shown in red with node sizes indicating their nodal efficiency values. The network shown here was constructed by averaging the WM connection matrices of each group and thresholded with the sparsity of 10%. The regions were mapped onto the cortical surface at the lateral view. The nodal regions are located according to their centroid stereotaxic coordinates. For the abbreviations of nodes, see Table 2.

For the control group, the identified hub nodes (15 in total, Figure 3 and Table 4) included 8 regions of the association cortex, 4 regions of the primary cortex, 2 regions of subcortical structures and 1 paralimbic region. The 17 hubs for the NMO group included 8 regions of the association cortex, 4 regions of the primary cortex, 4 regions of subcortical structures and 1 in the paralimbic region (Figure 3 and Table 4). Fourteen hub regions were the same for both groups, including bilateral precuneus (PCUN), bilateral precentral gyrus (PreCG), bilateral postcentral gyrus (PoCG), bilateral dorsolateral superior frontal gyrus (SFGdor), bilateral middle frontal gyrus (MFG), left medial superior frontal gyrus, bilateral putamen and right median cingulate gyrus. One hub region, the left inferior parietal gyrus (IPL), was present in the control group but not in the NMO group. The left middle occipital gyrus and bilateral thalami were identified as hubs in the NMO group but not in the control group. From the results, the hubs that we located for both groups were predominantly in regions of heteromodal or unimodal association cortex, which receive convergent inputs from multiple cortical regions [42], consistent with many previous studies [15], [16], [18], [26], [29].

**Table 4:** Hub Regions of WM networks in control and NMO groups.

Control			NMO		
Hubregions	Class	E_nodal/_mean	Hubregions	Class	E_nodal/_mean
PCUN.R	Association	1.58	PCUN.R	Association	1.45
PCUN.L	Association	1.56	PCUN.L	Association	1.40
PreCG.L	Primay	1.52	PreCG.R	Primay	1.40
PoCG.L	Primay	1.46	PreCG.L	Primay	1.37
PoCG.R	Primay	1.46	PoCG.R	Primay	1.37
SFGdor.R	Association	1.46	PoCG.L	Primay	1.36
PreCG.R	Primay	1.44	SFGdor.R	Association	1.33
MFG.L	Association	1.40	MFG.L	Association	1.32
DCG.R	Paralimbic	1.40	DCG.R	Paralimbic	1.31
SFGmed.L	Association	1.36	PUT.L	Subcortical	1.32
SFGdor.L	Association	1.35	SFGdor.L	Association	1.32
MFG.R	Association	1.32	MFG.R	Association	1.32
PUT.L	Subcortical	1.30	SFGmed.L	Association	1.30
IPL.L	Association	1.29	PUT.R	Subcortical	1.29
PUT.R	Subcortical	1.28	MOG.L	Association	1.28
			THA.L	Subcortical	1.24
			THA.R	Subcortical	1.24

The hub regions were identified if E_nodal_(i) was at least one SD greater than the mean nodal efficiency of the network (i.e., E_nodal_(i) > mean±SD). The hubs are sorted by the mean normalized nodal efficiency (divided by the mean of all nodes) in each group. The cortical regions are classified as primary, association and paralimibic [42].

#### Group differences in regional efficiency

**Figure 4:**
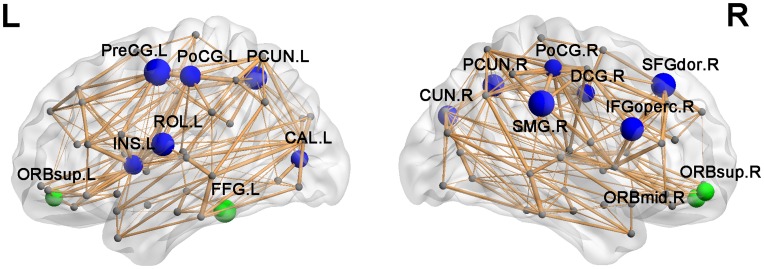
The brain regions with significant group differences in nodal efficiency between control and NMO groups at *p*<0.05 (uncorrected). The node sizes indicate the significance of between-group differences in the regional efficiency. The network shown here was constructed by averaging the WM connection matrices of all healthy controls and thresholded with the sparsity of 10%. The nodal regions are located according to their centroid stereotaxic coordinates. Nodes in blue showed the regions have reduced efficiency in NMO patients versus controls. Nodes in green showed the regions have increased efficiency in NMO patients versus controls. For the abbreviations of nodes, see Table 2.

Compared with healthy controls, 17 regions with significantly altered efficiency in NMO patients were identified at *p*<0.05 (uncorrected) (Figure 4 and Table 5). Most of these regions had reduced efficiency in NMO patients, including the left PreCG, right supramarginal gyrus (SMG), right dorsolateral superior frontal gyrus, right opercular part of inferior frontal gyrus, bilateral PCUN, left rolandic operculum, bilateral PoCG, right cuneus, left insula, right median cingulate and paracingulate gyri and left calcarine (CAL). Four regions were found with increased efficiency in NMO patients, including bilateral orbital part of superior frontal gyrus (ORBsup), right orbital part of middle frontal gyrus (ORBmid) and left fusiform gyrus (FFG).

**Table 5:** Brain regions with significant group effects in nodal efficiency between control and NMO groups.

Control >NMO				Control < NMO			
Regions	Class	*T*-value	*P*-value	Regions	Class	*T*-value	*P*-value
PreCG.L	Primary	−3.01	0.004	FFG.L	Association	2.59	0.013
SMG.R	Association	−2.92	0.005	ORBsup.R	Paralimbic	2.13	0.038
SFGdor.R	Association	−2.77	0.008	ORBmid.R	Paralimbic	2.10	0.041
IFGoperc.R	Association	−2.66	0.012	ORBsup.L	Paralimbic	2.05	0.046
PCUN.L	Association	−2.63	0.013				
ROL.L	Association	−2.63	0.013				
PCUN.R	Association	−2.50	0.016				
PoCG.L	Primary	−2.41	0.020				
CUN.R	Association	−2.34	0.023				
INS.L	Paralimbic	−2.23	0.030				
DCG.R	Paralimbic	−2.12	0.039				
PoCG.R	Primary	−2.08	0.043				
CAL.L	Primary	−2.03	0.048				

Note: The FN-weighted WM network for each participant was constructed under the threshold T = 3. The comparisons of nodal efficiency were performed between groups for each brain region. *p*<0.05 (uncorrected) was considered significant.

In order to fully account for the potential effect of non-specific white matter lesions, we identified and excluded the 5 patients with small, non-specific cerebral white matter lesions. All network analysis was repeated and results were not significantly different from the primary analysis which included all patients.

## Discussion

In this study, we constructed WM structural networks from DTI data to investigate potential alterations in network properties in patients with NMO compared to healthy controls. The NMO patients exhibited abnormal small-world network properties, as indicated by increased normalized path length, increased normalized clustering and increased small-worldness. Furthermore, largely similar hub distributions of the WM structural networks were observed between NMO and normal subjects. However, regional efficiency in several brain areas of NMO patients was significantly changed, and usually reduced, when compared with the healthy controls.

### Small-world Properties in the WM Networks in NMO

The human brain is a large, dynamic network system with an optimal balance between local specialization and global integration. In the present study, we characterized the small-world topology of the WM networks in both controls and NMO patients, using methods described in previous network studies based on various imaging techniques (e.g., structural MRI, functional MRI and EEG/MEG). Although the WM networks in NMO showed prominent small-world topology, we found patients with NMO had significantly altered global network organization relative to controls, exhibiting increased normalized path length, increased normalized clustering and increased small-worldness. Increased path length suggest reduced efficiency of parallel information transfer in the WM networks in NMO patients, while increased normalized clustering suggests a stronger local specialization. Given that the small-world connectivity model reflects an optimal balance between local specialization and global integration, the longer path length (λ), higher clustering (γ) and increased small-worldness in the NMO patients’ networks could indicate less optimal organization of the brain networks, possibly as a consequence of reorganization secondary to cortical injury.

### Distributed Regions with Altered Efficiency in NMO

In the present study, we observed several brain regions with reduced efficiency in NMO patients. For example, decreased efficiency was demonstrated in the PCUN, which is a hub region in the default-mode network (DMN), an intrinsic functional connectivity network extensively characterized in healthy volunteers. The DMN concept is derived from resting-state functional MRI, altered in Alzheimer’s disease and MS [43]–[45] and implicated in episodic memory processing [46], [47]. Similarly, the supramarginal gyrus, dorsolateral superior frontal gyrus, opercular part of the inferior frontal gyrus and the rolandic operculum are part of DMN, involving cognitive processing. Decreased nodal efficiency in these areas implies a pathological alteration in the DMN, potentially providing an explanation for cognitive impairment in NMO, as reported by Blanc et al. [48]. Recently, resting state functional MRI studies also demonstrated DMN alteration in NMO [49], supporting the findings of the current study.

Reduced regional efficiencies were also observed in the precentral and postcentral gyrus, which are key regions of the sensorimotor system. Previous fMRI studies showed abnormal movement-associated patterns of cortical activation in patients with cord damage of different origins [50]–[52] including patients with NMO [7], so that changes in these areas could be secondary to corticospinal tract damage in spinal cord lesions. Similarly, the cuneus (CUN), insula (INS) and calcarine (CAL) are visual processing centers, and the decreased regional efficiency of these brain areas could be related to the optic nerve damage in NMO. While it is also possible that all of the subtle alterations we have observed in different brain regions in NMO are in some way secondary to spinal cord and optic nerve pathology, we hypothesize that changes in DMN-associated WM regions are more likely secondary to relatively subtle cerebral pathology in NMO.

We also observed increased regional efficiency in the orbital parts of superior and middle frontal gyrus, and fusiform gyrus. These increased structural connections could indicate compensatory reorganization or recruitment [53].

### Convergent Evidence from Comprehensive Analyses

We reproduced our investigations by utilizing binary, FA-weighted and FN-weighted networks construction with different threshold values (T = 1,2,3,4,5). In each of these situations, we calculated the topological properties of brain networks for small-world evaluation and performed inter-group comparisons by statistical analyses. The results of these analyses showed that, in all the tested situations, prominent small-world characteristics were consistently observed in both control and NMO groups (Figures S1 and S2, Table 3). More importantly, abnormal small-world parameters of WM networks were consistently observed in the NMO group (Figures S1 and S2, Table 3). These comprehensive analyses provide additional evidence for the validity of our findings.

### White Matter Brain Networks of MS versus NMO

Recently, we investigated the WM networks of MS using a similar analysis method, and demonstrated decreased global and local efficiencies in patients [30]. In the present study, we demonstrate NMO-related changes in the WM structural brain networks. Although the loss of small-world properties was observed in both studies, there were distinct patterns. In MS patients, the WM brain networks tended to have much less connections (reduced network strength, global and local efficiencies) compared with controls. In contrast, in NMO, the structural brain networks exhibited a disrupted topological organization (abnormal small-world parameters but no changes in network strength and efficiencies). Further, we found a greater number of disrupted regions in MS compared to NMO. The likely explanation is that the extent of brain injury in MS is greater than NMO including the presence of T1 and T2 lesions in MS, but it is also possible that different lesion pathogenesis could contribute to the observed difference [2]. In order to confirm and extend these findings, future studies should ideally include new cohorts of both NMO and MS patients scanned using identical protocols on the same protocols on the same scanner.

### Methodological Issues

First, the present study used a suboptimal DTI sequence with six diffusion-encoding gradient directions and non-isotropic voxel size. Although, using similar scanning sequences, a recent study did report a high reproducibility of the WM network properties [15], our analysis should be reproduced with new patient and healthy control datasets derived from optimal scanning parameters. Second, we employed deterministic tractography to define the edges of the WM networks. This method has been used in previous DTI studies [15], [29]. However, the tracking procedure always stops when it reaches regions with fiber crossings and low FA values because of the “fiber crossing” problem [54], which might result in inappropriate loss of existing fibers. Other studies have proposed the use of probabilistic tractography to define the network edges [18], [20], or advanced diffusion imaging techniques such as diffusion spectral imaging [55] or high angular resolution diffusion imaging with Q-ball reconstruction of multiple fiber orientations [56], which could be helpful to address the issue. Third, we utilized DTI tractorgraphy to construct the WM networks. Brain networks can also be studied using structural and functional MRI data [24], [26], [57]. The combination of these multimodal MRI techniques would yield a more comprehensive understanding of how structural disruptions in brain networks are associated with functional deficits in patients with NMO, and should include detailed cognitive assessments.

### Conclusion

In the present study, we used diffusion tensor tractography and graph theoretical analyses to investigate NMO-related topological changes in WM structural networks. We found that, compared to controls, patients with NMO had abnormal small-world parameters in their brain networks. Moreover, we observed that regions with decreased efficiency were mainly distributed in the default-mode network, sensorimotor and visual systems. Although the NMO patients in this study had no discernible WM T2 lesions in the brain, we hypothesize that these findings provide additional evidence for subtle, widespread cerebral WM pathology in NMO.

## Supporting Information

Figure S1 **Global measures of WM structural networks were quantified in controls and NMO patients with different connectivity metrics (FA-weighted network).** The threshold (horizontal axis) determined the minimum number of streamlines that needed to interconnect a pair of nodes for a connection to be assumed. Data points marked with a star indicate a significant difference (*p*<0.05) between groups. Significant group effects in normalized clustering and small-worldness were observed for most thresholds considered.
(TIF)

Figure S2 **Global measures of WM structural networks were quantified in controls and NMO patients with different connectivity metrics (binary network).** The threshold (horizontal axis) determined the minimum number of streamlines that needed to interconnect a pair of nodes for a connection to be assumed. Data points marked with a star indicate a significant difference (*p*<0.05) between groups. Significant group effects in normalized clustering and small-worldness were observed for most thresholds considered.
(TIF)
