# Supplementary figures and images for: Altered Topological Organization of White Matter Structural Networks in Patients with Neuromyelitis Optica
Source: PLoS One. 2012 Nov 7;7(11):e48846. doi: 10.1371/journal.pone.0048846 (PMC3492259; doi:10.1371/journal.pone.0048846)

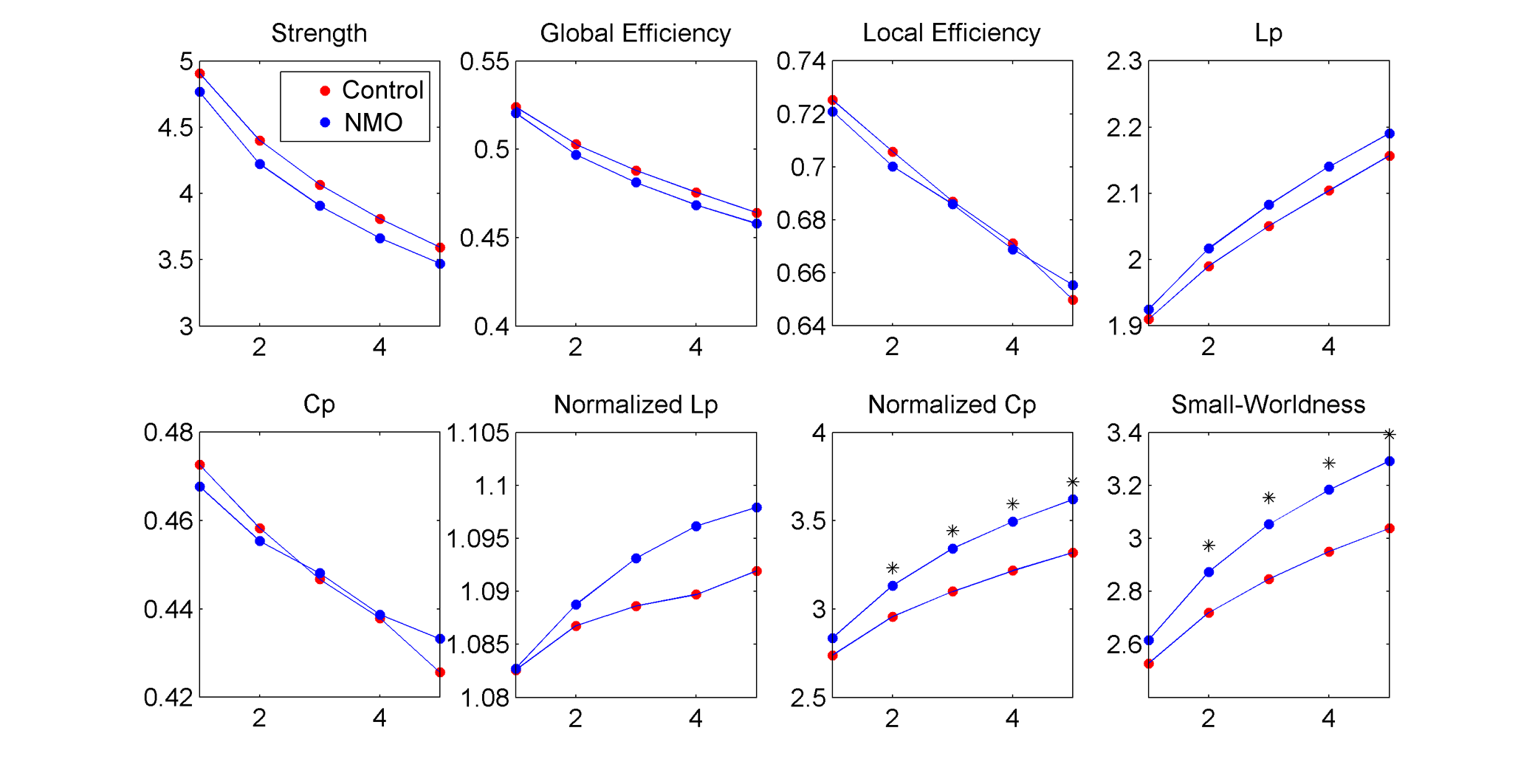

Supplement: Figure S1 — Global measures of WM structural networks were quantified in controls and NMO patients with different connectivity metrics (FA-weighted network). The threshold (horizontal axis) determined the minimum number of streamlines that needed to interconnect a pair of nodes for a connection to be assumed. Data points marked with a star indicate a significant difference (p<0.05) between groups. Significant group effects in normalized clustering and small-worldness were observed for most thresholds considered. (TIF) [file pone.0048846.s001.tif]

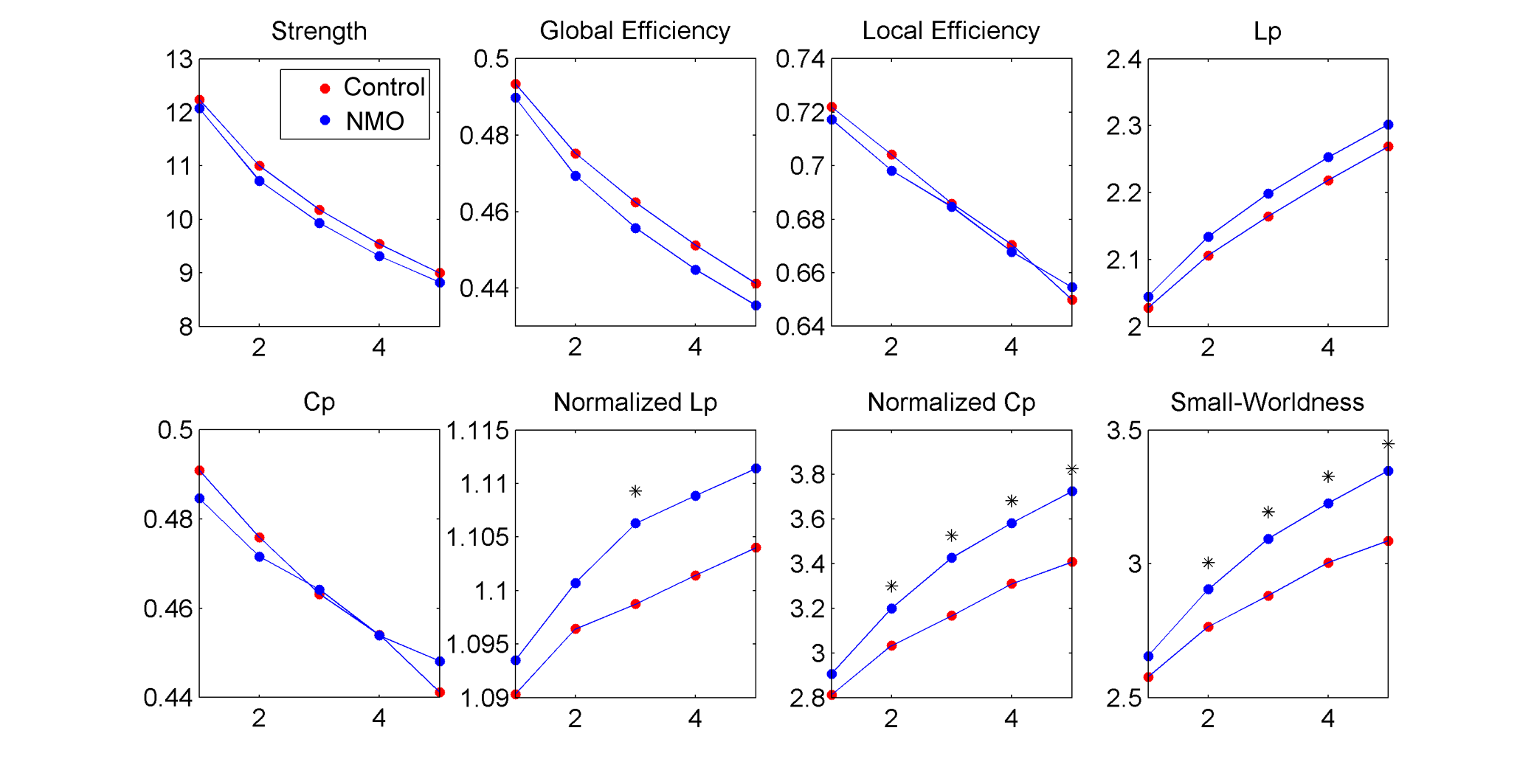

Supplement: Figure S2 — Global measures of WM structural networks were quantified in controls and NMO patients with different connectivity metrics (binary network). The threshold (horizontal axis) determined the minimum number of streamlines that needed to interconnect a pair of nodes for a connection to be assumed. Data points marked with a star indicate a significant difference (p<0.05) between groups. Significant group effects in normalized clustering and small-worldness were observed for most thresholds considered. (TIF) [file pone.0048846.s002.tif]
